# Nerve growth factor signalling in pathology and regeneration of human teeth

**DOI:** 10.1038/s41598-017-01455-3

**Published:** 2017-05-02

**Authors:** Thimios A. Mitsiadis, Henry Magloire, Pierfrancesco Pagella

**Affiliations:** 1 Orofacial Development and Regeneration, Institute of Oral Biology, Centre for Dental Medicine, Medical Faculty, University of Zurich, Zurich, Switzerland; 2 Institut de Génomique Fonctionnelle de Lyon, Ecole Normale Supérieure (ENS), Lyon, France

## Abstract

Nerve growth factor (NGF) is a key regulator of the development and differentiation of neuronal and non-neuronal cells. In the present study we examined the distribution of NGF and its low and high-affinity receptors, p75^NTR^ and TrkA respectively, in permanent human teeth under normal and pathological conditions. In intact functional teeth, NGF, p75^NTR^ and TrkA are weakly expressed in dental pulp fibroblasts and odontoblasts that are responsible for dentine formation, while the NGF and p75^NTR^ molecules are strongly expressed in nerve fibres innervating the dental pulp. In carious and injured teeth NGF and TrkA expression is upregulated in a selective manner in odontoblasts surrounding the injury sites, indicating a link between NGF signalling and dental tissue repair events. Accordingly, NGF and TrkA expression is strongly upregulated in cultured primary human dental mesenchymal cells during their differentiation into odontoblasts. Targeted release of NGF in cultured human tooth slices induced extensive axonal growth and migration of Schwann cells towards the NGF administration site. These results show that NGF signalling is strongly linked to pathological and regenerative processes in human teeth and suggest a potential role for this neurotrophic molecule in pulp regeneration.

## Introduction

Nerve growth factor (NGF) is the first identified member of a family of neurotrophic molecules that also comprises the brain derived neurotrophic factor (BDNF), neurotrophin-3 (NT-3), NT-4, and NT-6. All these molecules have essential roles in the fate, development, survival, outgrowth and maintenance of selected group of neurons^1–4^. NTs bind with either high or low affinity to a variety of specific receptors that are expressed on both neurons and non-neuronal target cells. The transmembrane glycoprotein receptor p75^NTR^ binds all NTs with low affinity^1, 4^, and its activation can induce either cell survival or apoptosis depending on the identity of the attached ligand^4–6^. The tyrosine kinases receptors TrkA, TrkB and TrkC also bind NTs with different affinities^1, 4^. The 140 kDa glycoprotein TrkA acts as high affinity receptor for NGF and its function is influenced by the concomitent presence of p75^NTR^, which can either inhibit or increase TrkA activation^1, 4, 7, 8^.

Apart their known functions in the nervous system, additional roles for NTs were suggested based on the expression patterns of p75^NTR^ and Trk receptors during organogenesis and differentiation of non-neuronal cells such as melanocytes, parafollicular cells of the thyroid gland, secretory and pillar cells of the cochlea, glomeruli of the kidney, somites, and odontoblasts and ameloblasts of the teeth^4, 9–15^. Moreover, in the last years the NTs/p75^NTR^ signalling was extensively studied in the context of stem cell function, and p75^NTR^ was identified as a marker of selected stem cell populations^2, 16^. NGF may act as a proliferative, differentiation, survival and apoptotic agent in non-neuronal cells, depending on its concentration and the presence of NTs receptors in these cells^1, 4, 17^.

Previous studies from our group and from others have demonstrated that NGF, p75^NTR^ and TrkA are expressed in the developing rodent and human teeth, thus suggesting that dental cell functions are dependent upon NTs signalling^13, 14, 18–24^. The tooth develops as a result of sequential and reciprocal interactions between the oral ectoderm and the cranial neural crest-derived mesenchyme^25, 26^. Differentiation of dental cells gives rise to the mesenchymal-derived odontoblasts that produce the dentine matrix and the epithelial-derived ameloblasts that form the enamel. In developing teeth, expression of NGF, p75^NTR^ and TrkA in mesenchyme correlates with cytodifferentiation events, while epithelial expression is linked to proliferative phenomena^13, 14, 20, 24^. *In vitro* studies have shown that NGF produced by dental pulp fibroblasts acts as a neuroattractive agent^27^, and therefore NGF could have a role in the attraction of neurons into the pulp chamber during odontogenesis^13^. Studies in rodents and dogs have shown that expression of NGF and p75^NTR^ increased after dental injury, suggesting additional roles for NGF in regenerative processes^18, 19, 28, 29^. Although all these findings illustrate the importance of NGF signalling in a variety of biological processes linked to tooth development, homeostasis and repair, only very few studies have been realized in human teeth to date. While these studies reported on the expression of TrkA^24^, p75^NTR^ ^22^, and *NGF* mRNA^23^ during odontogenesis, there is not yet data concerning their expression in adult human teeth under normal and pathological conditions.

The present study was conducted to analyze the *in vivo* expression of the NGF, p75^NTR^ and TrkA proteins in intact, carious and injured adult human teeth, as well as in cultured human dental pulp cells *in vitro*. Furthermore, we examined the effects of NGF on neuronal sprouting in an *in vitro* dental slice culture model.

## Results

### NGF, p75^NTR^ and TrkA expression in healthy human teeth

**Figure 1:**
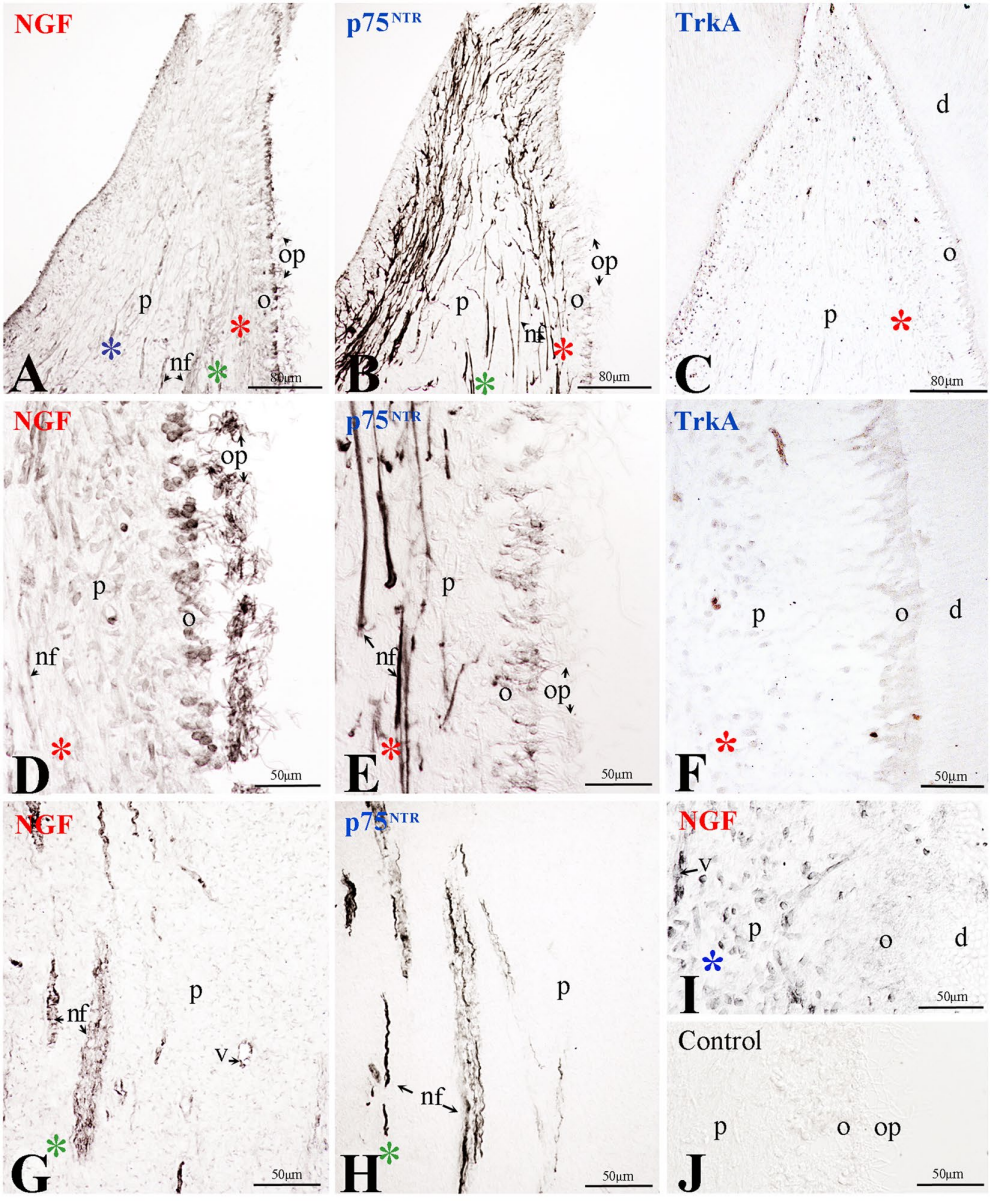
Expression of NGF, p75^NTR^ and TrkA in healthy human teeth. (**A**–**C**) Overview of NGF (**A**), p75^NTR^ (**B**) and TrkA (**C**) expression in the pulp of healthy teeth. (**D**,**G**,**I**) NGF, (**E**,**H**) p75^NTR^ and (**F**) TrkA expression in selected regions of healthy intact human teeth. (**J**) Negative control. Abbreviations: d, dentine; nf, nerve fibres; o, odontoblasts; op, odontoblastic processes; p, pulp; v, vessel. Asterisks in different colours in Fig. 1A–C indicate higher magnifications of the corresponding areas in Fig. 1D–I.

In the dental pulp of permanent intact teeth, very little, if not at all, NGF and TrkA immunoreactivities could be observed in decalcified specimens (Fig. 1C,F,I). In freshly extracted non-decalcified pulps, moderate NGF immunoreactivity was observed in odontoblasts and nerve-related cells (Fig. 1A,D,G), while p75^NTR^ staining was strong in nerve fibers and very faint in odontoblasts (Fig. 1B,E,H). The most intense NGF labelling was detected in the odontoblastic processes, while the staining was weaker in the odontoblastic bodies (Fig. 1D). In general, NGF staining was absent in dental pulp fibroblasts (Fig. 1D,G), although NGF immunoreactivity was seen sporadicly in some of these cells (Fig. 1I). In contrast, p75^NTR^ immunoreactivity was absent from all pulp fibroblasts (Fig. 1E,H). Immunostaining for NGF, p75^NTR^ and TrkA was not detected in the sections used as controls (Fig. 1J).

### NGF, p75^NTR^ and TrkA expression in carious human teeth

**Figure 2:**
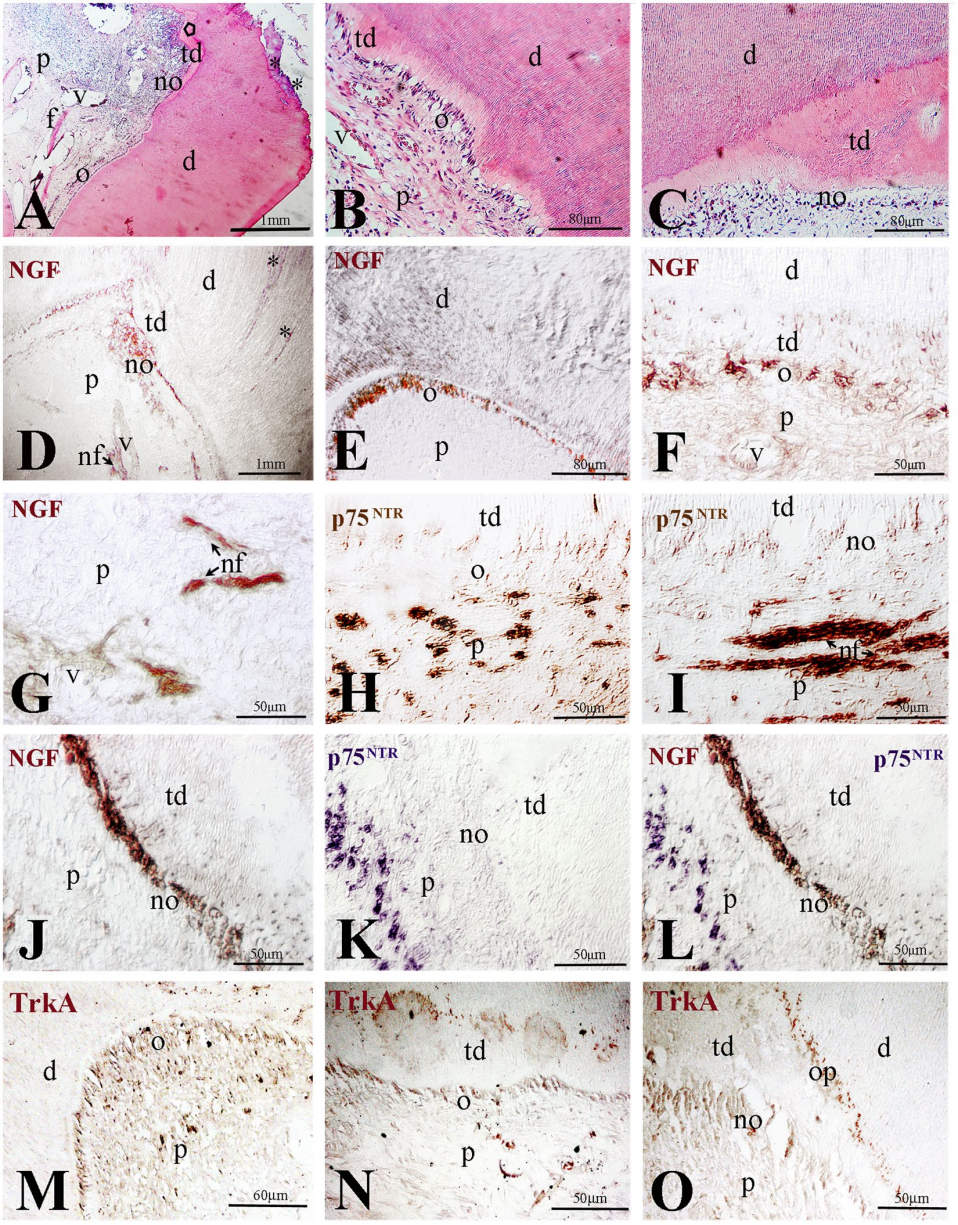
Expression of NGF, p75^NTR^ and TrkA in carious human teeth. (**A**–**C**) Histology of carious teeth. (**B**,**C**) higher magnifications of regions of production of tertiary dentine. (**D**–**F**) NGF expression (brown colour) in regions underlying the carious lesion (**D**), in proximity of the lesion (**E**), in odontoblasts producing tertiary dentine (**F**). (**G**) Expression of NGF in nerve fibres within the pulp. (**H**,**I**) p75^NTR^ expression (brown colour) in nerve fibres and vessels underlying newly formed odontoblasts. (**J**–**L**) Co-staining showing NGF (brown colour) and p75^NTR^ (violet colour) expression in the region underlying the carious lesion. (**M**–**O**) TrkA expression (brown colour) in odontoblasts facing the carious region (**M**) and in odontoblasts producing the tertiary dentin (**N**,**O**). Abbreviations: d, dentine; nf, nerve fibres; no, newly-formed odontoblasts; o, odontoblasts; op, odontoblast processes; p, pulp; td, tertiary dentine; v, vessel; Asterisks indicate the progression of the carious lesion in the dentine.

In teeth with carious lesions, the secretory activity of the odontoblasts is stimulated to produce tertiary dentine (Fig. 2A–C). Inflammatory events are often observed in the pulp of teeth with advanced carious lesions (Fig. 2A). In these case, odontoblasts beneath the bacterially infected dentine die and are replaced by newly differentiated odontontoblasts that form the tertiary dentine^30^. In decalcified carious teeth, NGF immunostaining was detected in both apoptotic and newly formed odontoblasts facing the decay (Fig. 2D,E,F,J,L). Notably, NGF expression could be observed also in nerve fibers and in perivascular regions within the pulp (Fig. 2G), where p75^NTR^ was also detected (Fig. 2I). Very weak p75^NTR^ expression was observed in odontoblasts, both in proximity and at a distance from the site of injury (Fig. 2H,I,K,L). TrkA staining was obvious in odontoblasts facing the carious lesion (Fig. 2M) as well as in odontoblasts that actively produce the tertiary dentine (Fig. 2N,O). TrkA immunoreactivity was detected in the bodies of the odontoblasts as well as in their processes (Fig. 2O). These results show that expression of both NGF and TrkA is strongly upregulated in odontoblasts and newly-formed odontoblasts in carious human teeth.

### NGF, p75^NTR^ and TrkA expression in permanent human teeth after cavity preparation

**Figure 3:**
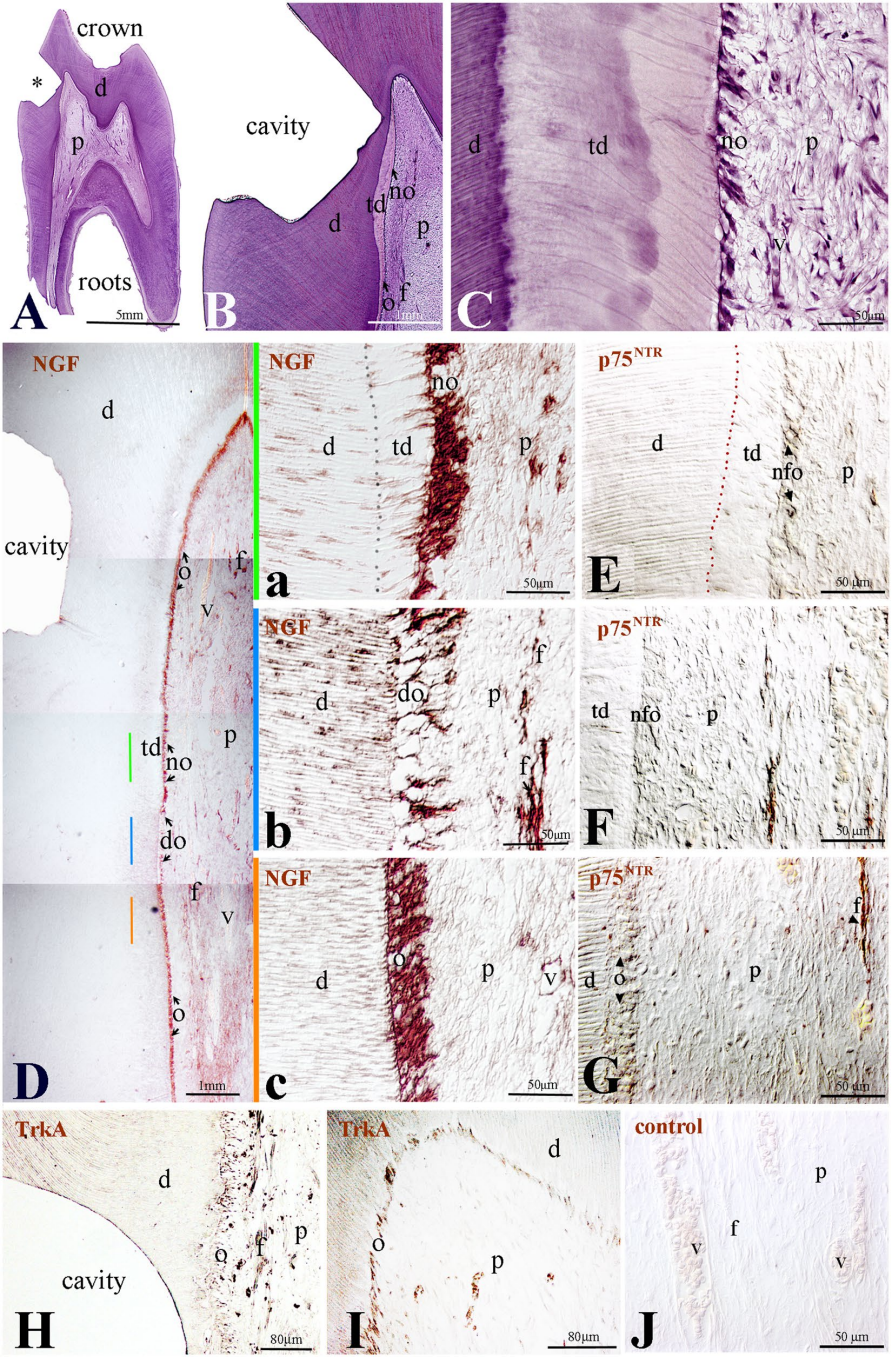
Expression of NGF, p75^NTR^ and TrkA in permanent human teeth after cavity preparation. (**A**–**C**) Histology of dental tissues after cavity preparation. (**D**) Overview of NGF staining (brown colour) in tooth after cavity preparation. (**D**(a–c)) Higher magnifications of D. (**E**) p75^NTR^ expression (brown colour) in newly formed odontoblasts underlying tertiary dentin. (**F)** p75^NTR^ expression in nerve fibres in proximity of tertiary dentin. (**G**) p75^NTR^ expression in odontoblasts (arrowheads) and in nerve fibres within the pulp. (**H**,**I**) TrkA expression (brown colour) in odontoblasts and nerve fibres adjacent to the cavity (**H**) and far away from the injury site (**I**). (**J**) Negative control. Abbreviations: d, dentine; do, dying odontoblasts; f, nerve fibres; no, newly-formed odontoblasts; o, odontoblasts; p, pulp; v, vessel. Asterisk indicates the cavity.

Injury caused by tooth cavity preparation invokes pulp responses that are accompanied by apoptotic events^30^. Apoptotic odontoblasts are substituted by newly-formed odontoblasts, which are aligned into a single cell-layer and elaborate the tertiary dentine (Fig. 3A–C). These events were not observed in other pulp areas that are located at a distance from the cavity preparation site (Fig. 3A,B). Nine weeks post-surgery, a thick layer of tertiary dentine was seen beneath the injury site (Fig. 3C). Strong NGF immunoreactivity was detected in the newly-formed odontoblasts that secrete the tertiary dentine matrix (Fig. 3D(a)), but also in apoptotic odontoblasts (Fig. 3D(b)) as well as in odontoblasts located far away from the cavity (Fig. 3c). NGF staining was mainly distributed in the bodies of the odontoblasts, while a weak staining was found in their processes (Fig. Da–c). Quite often, cavity preparations generate hyperemic conditions within the pulp that affect blood vessel physiology. NGF staining was observed in cells of the dilated blood vessels, as well as in few pulp fibroblasts and neurons in the proximity of the vessels (Fig. 3D(a–c)). Strong p75^NTR^ expression was observed in nerve fibres, while weak expression was detected in odontoblasts (Fig. 3E–G). Intense TrkA labelling was observed in odontoblasts located near or far away from the cavity (Fig. 3H,I), while the staining was very weak in dying odontoblasts beneath the cavity (Fig. 3H). No staining was detected in control sections (Fig. 3J).

### NGF, p75^NTR^ and TrkA expression in human dental pulp cells *in vitro*

**Figure 4:**
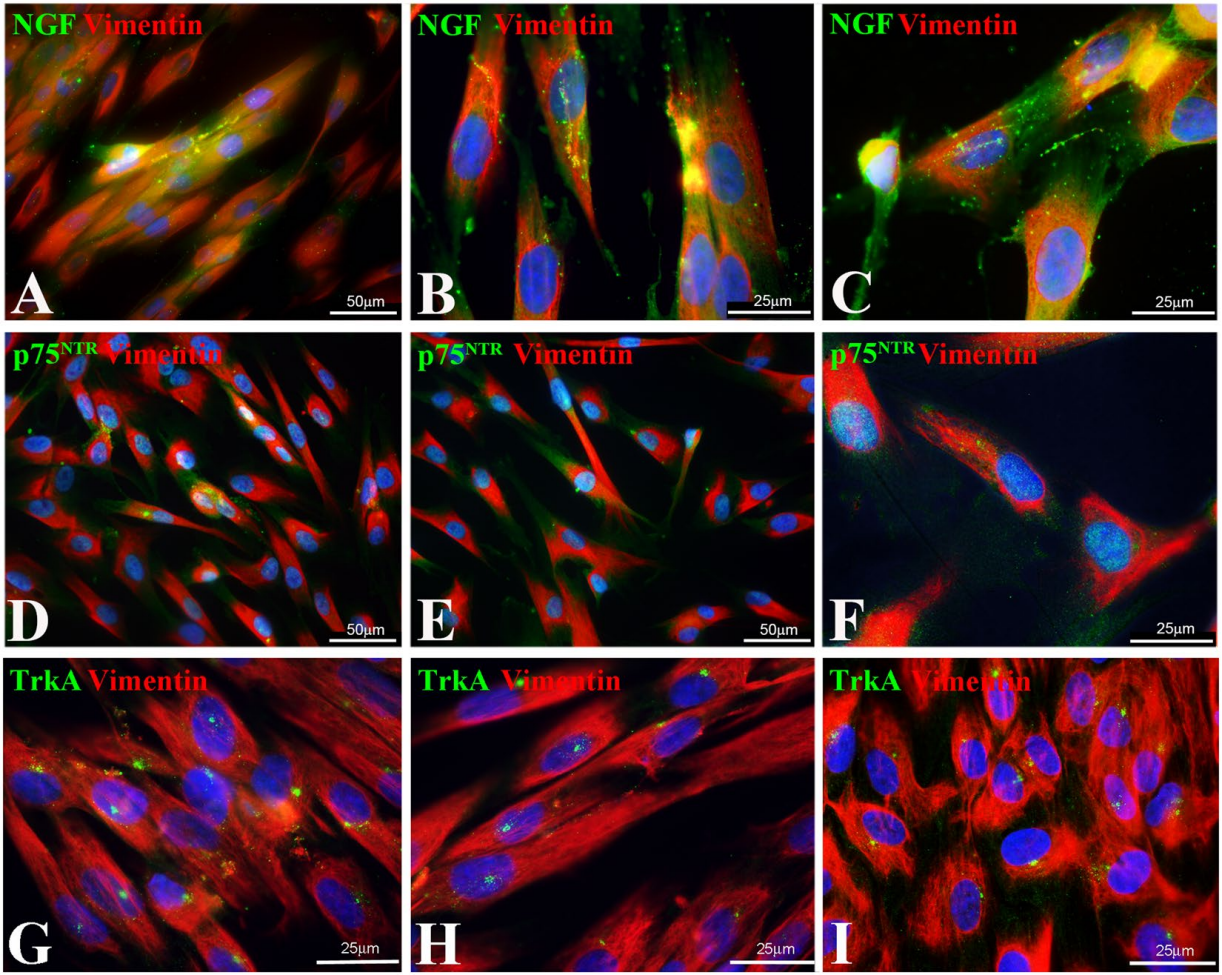
Expression of NGF, p75^NTR^ and TrkA in combination with vimentin in human dental pulp stem cells (hDPSCs). (**A**–**C**) NGF, (**D**–**F**) p75^NTR^ and (**G**–**I**) TrkA expression in hDPSCs.

**Figure 5:**
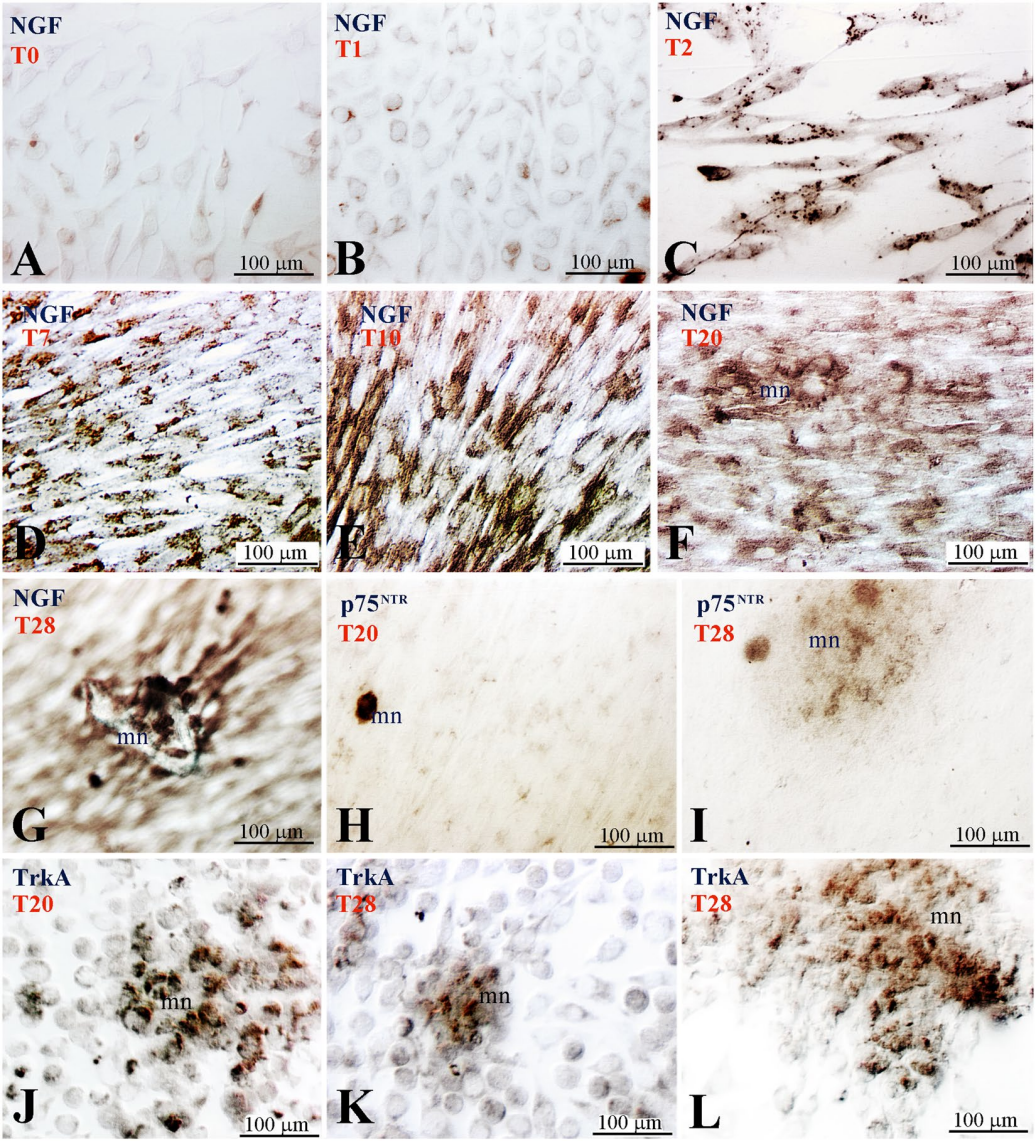
Expression of NGF and p75^NTR^ in human pulp cells cultured in the presence of β-glycerophosphate. (**A**) NGF expression at time 0 (T0). (**B**–**G**) NGF expression cultured in mineralizing conditions at successive time points (T1–T28). (**H**,**I**) p75^NTR^ expression in cells forming a mineral nodule (T20–T28). (**J**–**L**) TrkA expression in cells forming the mineral nodules (T20–T28). Abbreviation: mn, mineralized nodule; T1, one day of culture.

In order to better understand the role of NGF signalling in homeostasis and repair of human dental pulp we have analysed NGF, p75^NTR^ and TrkA expression in primary dental pulp cell cultures and in dental pulp stem cells (hDPSCs). In cultures of hDPSCs, only a very tiny group of cells expressed NGF (Fig. 4A–C), p75^NTR^ (Fig. 4D–F) and TrkA (Fig. 4G–I). We then studied the expression of these molecules in dental pulp cells cultured in mineralizing conditions that require the presence of ß-glycerophosphate (Fig. 5). All dental pulp cells cultured in presence of ß-glycerophosphate for different periods of time up to 4 weeks exhibited NGF immunoreactivity (Fig. 5B–G). The strongest NGF staining was observed in cells forming the mineralized nodules (Fig. 5F,G). p75^NTR^ staining was weak in cultured pulp cells but its expression increased in cells producing mineral matrix (Fig. 5H,I). Intense TrkA labelling was observed in cultured pulp cells forming the mineralized nodules (Fig. 5J–L).

### TGFβ1 deregulates NGF expression in human dental pulp

**Figure 6:**
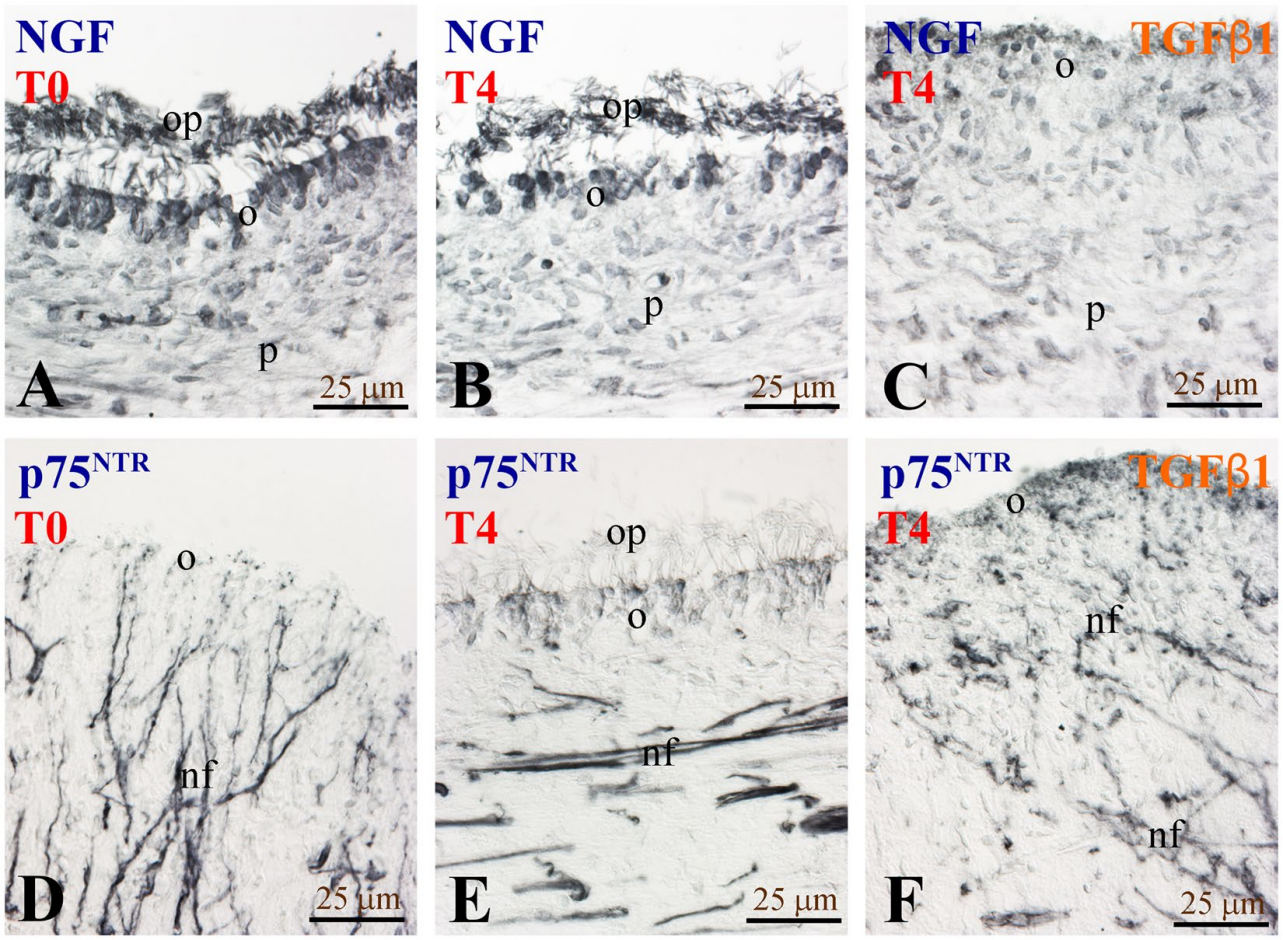
Effects of TGFβ1 on NGF and p75^NTR^ expression in cultured tooth slices *in vitro*. (**A**) NGF expression at T0 (starting point, time 0). (**B**) NGF expression at T4. (**C**) NGF expression at T4 upon treatment with TGFβ1. (**D**) p75^NTR^ expression at T0. (**E**) p75^NTR^ expression at T4. (**F**) p75^NTR^ expression at T4 upon treatment with TGFβ1. Abbreviations: nf, nerve fibres; o, odontoblasts; op, odontoblastic processes; p, pulp.

Based on the observation than NGF expression is upregulated in differentiating dental pulp cells, it is possible that TGFβ1 plays a role in this process, since it has been shown that TGFβ1 is involved in odontoblast differentiation^31^. To test this hypothesis, we followed the expression of NGF and p75^NTR^ in presence or absence of recombinant TGFβ1 protein in thick dental explants cultured *in vitro* for 4 days (Fig. 6). In absence of TGFβ1, weak NGF immunoreactivity was found in the odontoblasts from the first day of culture and this expression was maintained for the whole culture period (Fig. 6A,B). The presence of TGFβ1 did not affect NGF expression in odontoblasts (Fig. 6C). In absence of TGFβ1, p75^NTR^ immunoreactivity was detected in the odontoblasts and in nerve fibers for the whole culture period (Fig. 6D,E). Addition of TGFβ1 in the culture medium did not affect p75^NTR^ expression in both odontoblasts and nerve fibres (Fig. 6F). However, the structure of the nerve fibers appeared disorganized and fragmented (compare Fig. 6D and E with Fig. 6F).

### Administration of NGF in dental tissues induces axonal sprouting and migration of Schwann cells

**Figure 7:**
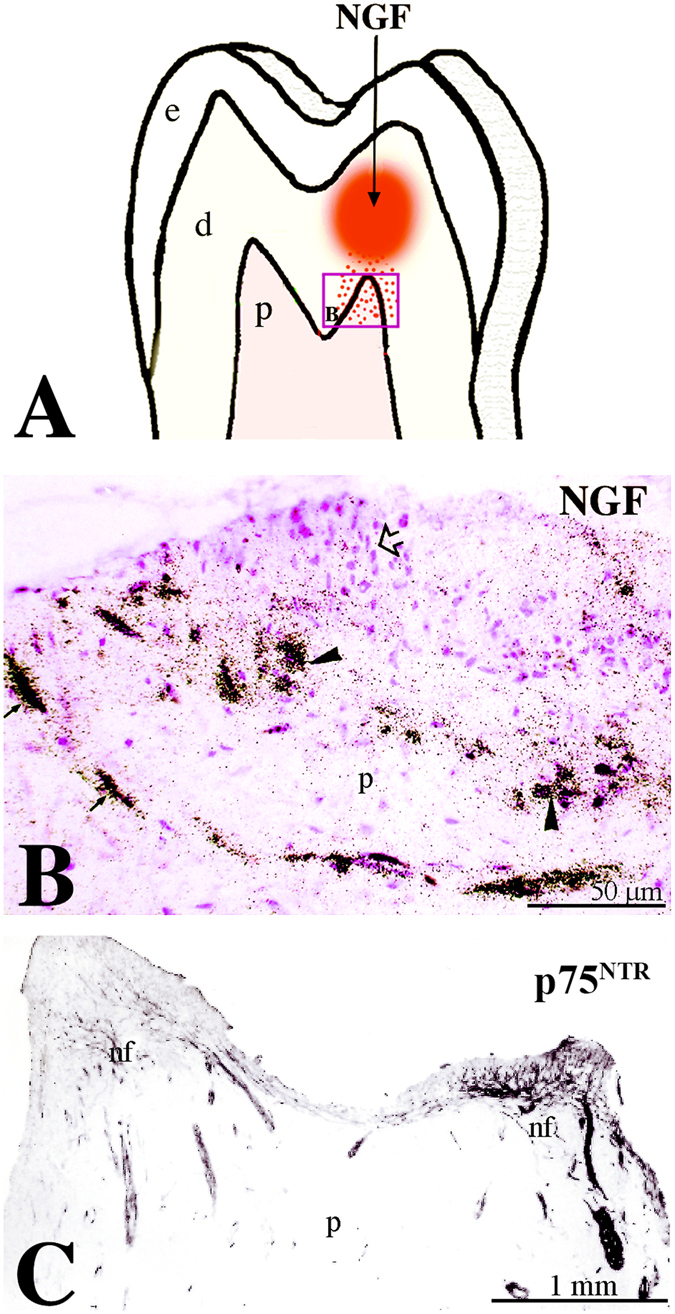
Administration of radioactive NGF (^125^I-NGF) induces nerve growth towards the release site. (**A**) Schematic representation of the experimental approach for ^125^I-NGF infusion. (**B**) Detection of ^125^I-NGF diffusion in the pulp. (**C**) p75^NTR^ expression marking neurites growth towards the site of NGF infusion. Abbreviations: nf, nerve fibres; p, pulp.

**Figure 8:**
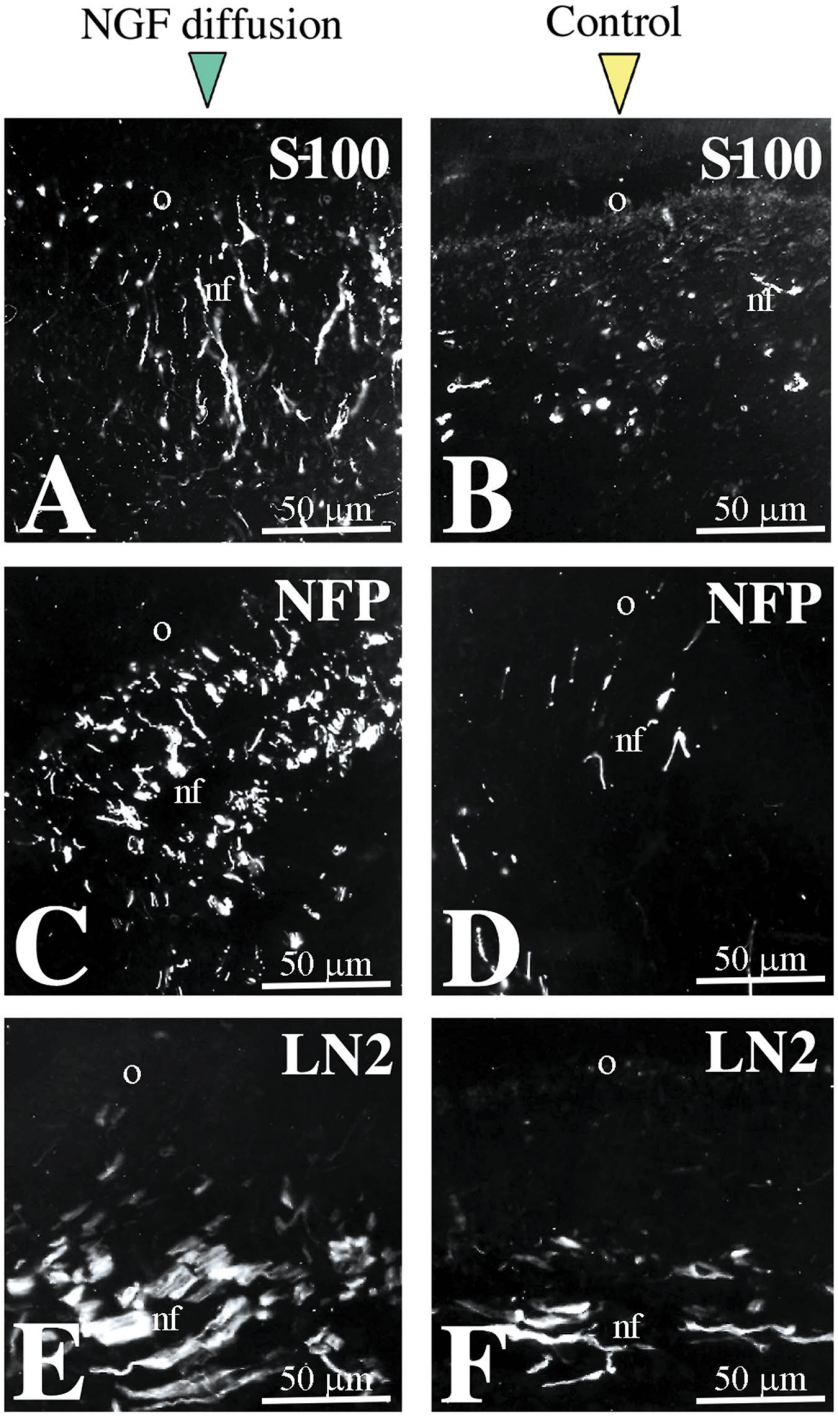
NGF administration induces growth of axons and migration of glial cells. (**A**,**B**) S-100 staining marking glial cells in NGF-treated (**A**) and control (**B**) pulps. (**C**,**D**) Neurofilament (NFP) staining marking axons in NGF-treated (**C**) and control (**D**) pulps. (**E**,**F**) Laminin-α2 (LN2) expression in NGF-treated (**E**) and control (**F**) pulps. Abbreviations: nf, nerve fibres; o, odontoblasts.

Since NGF has a pivotal role in axonal guidance, growth and sprouting we wish to know whether it is able to affect neurites within dental pulp and atract them towards the injured/pathologic area where NGF upregulation was seen *in vivo*. For this purpose we used an *in vitro* human dental pulp slice model, where we applied radioactive NGF (^125^I-NGF) within the dentine via a propylene tube (Fig. 7A). ^125^I-NGF diffused through the dentineal tubules and after seven days of culture could be detected within the dental pulp (Fig. 7B). In correspondance to the region where ^125^I-NGF was released, we observed a significant increase in the expression of p75^NTR^ within the dental pulp, when compared to the non treated pulp horn (Fig. 7C). Furthermore, a considerable increase of S-100 (a specific marker for Schwann cells) (Fig. 8A,B) and NFP (a general marker of nerve fibres) (Fig. 8C,D) staining was observed at the sites of ^125^I-NGF release. Interestingly, labelling with laminin-α2, which promotes axonal growth^32^, also increased at the site of NGF delivery (Fig. 8E,F).

## Discussion

Tooth development, homeostasis and pathology are controlled by a complex network of molecules, which guarantee tissue integrity and function^26, 33^. Among these molecules, neurotrophic factors are important for tooth innervation as well as for cytodifferentiation and mineralization events. Our recent studies have shown that NGF and its receptors p75^NTR^ and TrkA are expressed in the developing human teeth^24^. More specifically, these proteins are distributed in differentiating odontoblasts secreting the mantle dentine matrix, illustrating a close correlation between the appearance of NGF-related molecules and the process of odontoblast differentiation. While numerous studies are undertaken in rodents to understand the role of NGF signalling in injured teeth, there are not equivalent studies in humans. Our results revealed that NGF, p75^NTR^ and TrkA are expressed in human functional intact teeth as well as in carious and injured teeth. However, there were noticeable differences in the expression patterns of these molecules. In the pulp of healthy adult teeth, NGF is faintly expressed in functional odontoblasts, while expression of both p75^NTR^ and TrkA in these cells is very weak. A number of dental pulp fibroblasts and cells correlated with neuronal structures express NGF, whereas p75^NTR^ is distributed to the dense neuronal plexus of the pulp. The same expression patterns have been observed previously in rodent teeth^13, 14, 20^, illustrating a close correlation between NGF expression and odontoblast differentiation and function. Continuous expression of NGF by odontoblasts of permenant human teeth indicates that this molecule, in addition to its neuroattractive role, may be implicated in the processes of dentine matrix synthesis and mineralization. This hypothesis can be reinforced by the fact that NGF is strongly expressed in differentiating odontoblast-like cells that form mineralization nodules, which also express TrkA and low levels of p75^NTR^ in cultures of human dental pulp cells *in vitro*.

In contrast to the previous findings in developing human teeth showing no p75^NTR^ immunoreactivity in dental pulp^22, 24^, we found weak p75^NTR^ labelling in the odontoblastic layer suggesting that odontoblasts express also the p75^NTR^ receptor. It is equally possible that this staining may be due to the existence of small nerve endings at the odontoblastic layer that intermingle with the odontoblasts. However, the presence of p75^NTR^ in pulp cells forming the mineralization nodules *in vitro* indicates that this receptor, acting together with TrkA, could be also involved in cytodifferentiation events. Further studies using electron microscopy will be needed to elucidate questions about p75^NTR^ expression in odontoblasts.

The capacity of the pulp to resist and repair injuries is fundamental for maintaining tooth integrity and homeostasis. Although in the pulp of healthy teeth cell divisions and dentine matrix secretion by odontoblasts are reduced, these processes are stimulated in carious lesions or traumatic injuries^33, 34^. The cellular responses to various degrees of tooth injury are wide-ranging and target to prevent bacterial invasion into the pulp chamber, inhibit the lesion, and induce wound healing. Therefore, all the dental pulp tissue is involved in this reparative process and not only the areas neighbouring the dental pathologic territory. In mild injuries such as slow progressing caries, the transiently damaged odontoblasts do survive, their morphology remains normal and their activity is stimulated leading to the production of a tertiary dentine called reactionary dentine^33, 34^. In these conditions, integrity of the injured odontoblasts is ensured by increased protein synthesis that targets to the reorganization of their cytoskeleton^35^, which is an important process during wound healing^36^. For example, activation of nestin expression in odontoblasts facing the carious decay has been associated with odontoblast cytoskeletal re-organization^37^. NGF and TrkA are also upregulated in the odontoblasts beneath the carious front, suggesting that NGF may play a role in this process. Indeed, numerous studies have linked NGF signalling with reorganization of the cytoskeleton in a variety of neuronal and non-neuronal cell types^38^, inclunding odontoblasts^39^. Therefore, an additional function for NGF in tertiary dentine matrix synthesis and secretion should not be excluded.

Fast progressing caries and operative procedures involving the dentine lead to the apoptosis of the primary odontoblasts^30, 33^. Formation of tertiary dentine (also called reparative dentine) at the pathologic site results from the proliferation and recruitment of dental pulp stem cells and their differentiation into a second-generation of odontoblasts^30, 40^. In pathological conditions, NGF can exert a variety of effects that promote cell survival and/or apoptosis^4^. Expression of NGF and TrkA in both apoptotic and newly-formed odontoblasts indicates that NGF signalling may be involved in apoptotic, cytodifferentiation and secretory events after severe tooth injury. NGF and TrkA are also activated in odontoblasts far away from the injury site, indicating their interconnectivity in reparing damaged dental tissues. It is worth noting that NGF expression is also activated in severe pathologies of broad interest such as injuries of the central nervous system and tumorigenic conditions within and outside the nervous system^2^. In dental injuries of strong intensity, the blood vessels of the pulp dilate and inflammatory responses are triggered in order to limit pulp damage. NGF is expressed in cells related to these vascular structures, thus indicating a correlation between NGF upregulation and pulp inflammatory events, similarly to what is known in the nervous system^41^.

It has been shown that the TrkA has a pivotal role in the pain perception in several organs, including teeth^24, 42, 43^. In humans, *NTRK1* mutations (the gene coding for the TRKA protein) lead to pain insensitivity^44, 45^. The observed upregulation of NGF and TrkA following tooth injury suggests that NGF signalling is involved in pain perception after dental injury. TrkA inhibition could be a valuable therapeutic tool to reduce dental pain perception. Indeed, NGF signalling gained great attention as targets for treatments against cancer-associated pain during the last years^46^.

Signalling molecules that are expressed by dental pulp cells could play a role in tooth repair events^33^. A variety of *in vitro* findings and genetic studies in mice have demonstrated the importance of Transforming Growth Factor-beta1 (TGFβ1) in dental tissue homeostasis and regeneration^33, 36, 47, 48^. *In vitro* studies using human dental tissues have shown that TGFβ1 induces proliferation and migration of pulp fibroblasts at the injury area as well as odontoblast differentiation^31^. Our results show that NGF is also upregulated in differentiating odontoblast-like cells in cultured human dental pulp cells. It is thus possible that NGF may interact with TGFβ1 to stimulate reparative events within the pulp.

Several studies showed that p75^NTR^ is expressed by a number of stem cell populations of different origins^2^, including dental pulp stem cells^49^. Our results show that p75^NTR^ is upregulated only in the sub-odontoblastic cell layer in human tooth slices after TGFβ1 administration, thus suggesting that the NTFs/p75^NTR^ signalling regulates the fate of odontoblast progenitors. Surprisingly, the organization of nerve fibres within the pulp was disturbed in the presence of TGFβ1.

Sprouting nerves may have stimulatory roles in the healing process by delivering neuropeptides and other substances to the wounded site^50^. The sensory nerve endings are perceptive to signals released during dental injury, resulting in nerve sprouting at the site of the lesion. It has been already established that NGF is one of the wound-induced signals controlling neuronal regeneration and sprouting^51^. The occurrence of sensory nerve outgrowth in relation to pulp injury has been already described in rodent teeth^18, 52^. Similar effects have been observed in studies using transgenic mice overexpressing NGF^53, 54^ as well as on NGF-infused brain^55^ and skin^56^. Our *in vitro* study of NGF release through the dentinal tubules of human tooth slices clearly demonstrates a localised outgrowth and sprouting of pulp nerves towards the NGF-releasing source. However, such neuro-attractive phenomenon could not be observed *in vivo* after cavity preparation, most probably because of the slow process of neuronal regrowth in humans and the much lower quantities of NGF secreted by odontoblasts when compared to the higher doses of administrated NGF *in vitro*. Schwann cells of the pulp, which are characterised by the expression of the p75^NTR^ and S-100 proteins, are involved in the process of nerve sprouting. These cells produce laminin-α2 that creates a favourable environment for axonal regrowth^32, 57^ and odontoblast differentiation^58^. The enrichment with Schwann cells at the site of NGF release suggests that this neurotrophic molecule might have an additional chemotrophic effect on glial cells by activating p75^NTR^ expression, as it has been demonstrated in other models of peripheral nerve regeneration^59, 60^. It is tempting to hypothesize that, upon injury in human teeth, Schwann cells can be reprogrammed to form odontoblasts, as it has been recently shown for glial cells of incisors in rodents^61^. Taken together, the above-mentioned observations indicate that NGF signalling is important for the recruitment of specific dental pulp cell populations and their subsequent differentiation into odontoblasts upon tooth injury in humans. A possible involvement of other neurotrophic factors (i.e., BNDF, NT-3, NT-4, NT-6) in these processes has not to be excluded.

In conclusion, the present results demonstrate that NGF signalling in dental pulp is associated with odontoblast differentiation, dentine matrix synthesis and neuronal attraction, and suggest that NGF plays a crucial role in regenerative events of pathological and injured human teeth.

## Materials and Methods

### Collection of human pulps and teeth

All procedures were performed in accordance with the current guidelines. Cavity preparation and tooth extraction were performed by professional dentists. Experimental procedures involving the use of human specimens were approved by the Kantonale Ethikkommission of Zurich (reference number 2012-0588). All procedures were performed after obtainance of informed written consent from all patients.

### Materials

Preparation, purification, and characterization of polyclonal anti-NGF antibodies have been described previously^13, 20^. Affinity purified mouse anti-human p75^NTR^ monoclonal antibody (20.4) was the kind gift of Dr E.M. Johnson Jr. and Dr C. Osborn (St. Louis, USA). The purification and characterization of the 20.4 antibody, which recognizes the human p75^NTR^, has been already described^62, 63^. Preparation and characterization of the rabbit anti-NGF antiserum has been already described^13^. This antiserum specifically identifies NGF in immunohistochemistry and in Western blots and does not cross react with other known neurotrophins. The rabbit anti-human/mouse/rat TrkA monoclonal antibody was purchased from Abcam (ab76291 – Cambridge, UK). Murine 2.5S NGF was obtained from Promega Corportation (Madison, WI, USA). Vector Vectastain ABC kit was purchased from Biosys (Compiègne, France). For the preparation of the culture media, all materials were purchased from Gibco BRL (Life Technologies Inc., NY, USA). Other chemicals were obtained from Sigma (St. Louis, MO, USA).

For cultures, Minimum Essential Medium (MEM) was supplemented with 10% fetal bovine serum, 2 mM glutamine, 100 UI/mL penicillin, 100 µg/mL streptomycin (Biowhittaker, Gagny, France), and 0.25 µg/mL amphotericin B (Fungizone®).

### Tissue preparation

Freshly extracted teeth used in this study comprised: a) ten premolars of 11–15 years old adolescents, b) ten intact and ten carious teeth (molars) of 40–45 years old patients.

For studies on injured dental tissues, cavities were prepared in intact first premolars scheduled for extraction at the Dental Care Center of Marseille. The pulp chambers were not exposed during the preparation of the cavities. The teeth were extracted after a post-operative interval of 9 weeks with the patients informed consent. Cavities were prepared in the flank of the crown (Fig. 3A) first by means of an intermittent application of an Airotor with water coolant to remove the enamel. Cavities 2–3 mm wide and 1.3–1.8 mm deep were then cut into the tooth dentine with a bur using the least possible pressure. The walls of the cavities were immediately conditioned with a 3% hydrogen peroxide solution and dried with a light stream. The cavities were then restored with the calcium hydroxide product Dycal (Dentsplay, USA).

Extracted teeth were fixed in 10% neutral-buffered formalin for 24 hours, demineralized in sodium formiate for 21 days, and then embedded in paraffin wax. Thereafter teeth were serially sectioned (6 µm thick sections) and processed for immunohistochemistry. For the detection of extremely low levels of NGF we proceeded with immediate pulp extraction from intact healthy teeth, followed by 10% neutral-buffered formalin fixation for 24 hours and embedded in paraffin without prior decalcification.

### Cultures of human dental pulp cells

Immediately after extraction of third molars from 17 years old patients, teeth were swabbed with 70% (v/v) alcohol. Teeth were then washed with sterile PBS and transferred into a laminar flow tissue culture hood in order to perform the rest of the procedures under sterile conditions. Dental pulps were gently removed with forceps, minced with scalpels, and rinsed with PBS. After mincing, each tooth pulp explant was cultured in 100-mm diameter culture dishes (Becton Dickinson Labware, Lincoln Park, NJ, USA) in MEM medium supplemented with 2 mM ß-glycerophosphate (Sigma Chemical Co., St Louis, USA). The explants were cultured at 37 °C in a humidified atmosphere of 5% CO_2_, 95% air and the culture medium was changed every other day. Confluent cultures were collected by trypsinization (0.2% trypsin and 0.02% EDTA). The cells were plated at 3 × 10^3^/cm^2^ on tissue culture treated 8-chambers glass slides (Becton Dickinson Labware, Lincoln Park, NJ, USA). After 4 weeks of culture, cells were fixed with 70% ethanol for one hour at 4 °C and processed for immunohistochemistry.

### Cultures of human dental pulp stem cells

Human dental pulp stem cells (hDPSCs) were isolated from the dental pulp of extracted impacted wisdom teeth of healthy patients as previously described^64^. The dental pulps were enzymatically digested for one hour at 37 °C in a solution of collagenase (3 mg/mL; Life Technologies Eυρoπε BV, Zug ZG, Switzerland) and dispase (4 mg/mL; Sigma-Aldrich Chemie GmbH, Buchs SG, Switzerland). A filtered single-cell suspension was plated in a 40 mm Petri dish with hDPSC growth medium containing DMEM/F12 (Sigma-Aldrich Chemie GmbH, Buchs SG, Switzerland) with 10% fetal bovine serum (FBS) (PAN Biotech GmbH, Aidenbach, Germany), 1% penicillin/streptomycin (P/S) (Sigma-Aldrich Chemie GmbH, Buchs SG, Switzerland), 1% L-glutamine (Sigma-Aldrich Chemie GmbH, Buchs SG, Switzerland), and 0.5 μg/ml fungizone (Life Technologies Europe BV, Zug ZG, Switzerland) after washing away the enzyme solution. Cells were passaged at 80–90% confluence^64^. Osteogenic, chondrogenic and adipogenic differentiation potential of hDPSCs was tested via standard differentiation protocols^65^.

### *In vitro* cultures using human tooth slices

Freshly extracted third molars were sliced in 750 µm thick sections as described previously^66^. Selected slices were washed and the dentine carefully dried by means of a sterile paper. A small propylene tube was glued to the dentine to allow diffusion of the factor-containing solution through the dentineal tubules as described previosly^67^. Tubes were filled only once with either 50 µL serum free medium containing NGF (50 ng/mL) or TGFβ1 (5 ng/mL) and the slices were cultured from 4 (for TGFβ1 administration) to 7 (for NGF administration) days. After culture, thick slices were rinsed in PBS, fixed in 4% paraformaldehyde (PFA)-PBS solution for 30 min. Some of the dental slices were equilibrated with 30% sucrose-PBS at 4 °C overnight, and then were embedded in Tissue-Tek OCT (Miles Lab., Elkhart, USA) and frozen in a liquid nitrogen/isobutane bath. 14 micrometer cryostat sections were mounted on slides and stored at −20 °C until staining.

### Immunostainings

Immunoperoxidase staining on sections was performed as previously described^13, 20^. Briefly, the sections were first deparaffinized, treated with 0.4% pepsin, exposed to a 0.3% solution of hydrogen peroxide in methanol, and then were incubated overnight at 4 °C with primary antibodies diluted in PBS/0.2% BSA. The mouse anti-human S-100 and p75^NTR^ primary antibodies (Boehringer Mannheim, Germany) were used at dilution 1:20 and 1:40 respectively. The polyclonal anti-NGF antibody was used at dilution 1:300, the monoclonal antibody 20.4 was diluted 1:200 to 1:1000 and the anti-TrkA antibody was diluted 1:70. Mouse anti-neurofilament proteins (NFP; Zymed labs, San Francisco, CA, USA) and anti-human laminin-α2 (merosin) (Chemicon International, Temecula, CA, USA) were diluted 1:50 and 1:2500, respectively. Peroxidase was revealed by incubation with 3.3-diaminobenzidine tetrahydrochloride (DAB) reaction solution. After staining the sections were mounted with Eukit. In control sections the primary antibodies were ommited.

For immunohistochemistry on cultured dental pulp cells, the incubation with primary antibodies was performed overnight at 4 °C after permeabilizion of cells for 15 min with 0.5% Triton X-100 in PBS. Peroxidase was revealed by incubation with 3-amino-9-ethylcarbazole (AEC) reaction solution and then the slides were mounted with Aquamount (BDH, Gurr, England). Controls were performed by incubations with unrelated primary antibodies.

For immunohistochemistry on thick slices, after blocking with 10% normal goat serum (NGS) in PBS for 30 min, slides were incubated in primary antibodies diluted in PBS overnight at room temperature, then extensively washed in PBS and incubated with goat anti-rabbit or sheep anti-mouse-IgG peroxidase conjugate diluted 1:50 (Sanofi Diagnostics Pasteur, France) and routinely subjected to the DAB (3-3′diaminobenzidine) procedure.

For immunofluorescence identification of S-100, NFP and laminin-α2 antigens, cryosections were incubated with primary antibodies and counterstained with a 1:70 dilution of anti-rabbit IgG FITC conjugate (Sanofi Diagnostics Pasteur, France). Thereafter sections were washed extensively and sealed with glycerol-phosphate buffered saline before examination with a Zeiss microscope. Negative controls were carried out with conjugates alone in place of the primary antibodies, the other steps remaining unchanged. For immunofluorescence identification of NGF and p75^NTR^ antigens on hDPSCs, cells were fixed in 4% PFA, washed extensively with PBS, blocked with PBS/2% BSA/0.5% Tween-20 and then incubated for 2 hours at room temperature with primary antibodies (polyclonal rabbit anti-NGF 1:100, monoclonal anti-p75^NTR^ 1:100, monoclonal anti-vimentin 1:300, monoclonal anti-TrkA 1:70) diluted in PBS/2% BSA/0.5% Tween-20. Samples were then washed extensively and incubated with secondary antibodies (alexa 488-conjugated anti-rabbit 1:250, alexa 568-conjugated anti-mouse 1:250; Life Technologies/Invitrogen) diluted in blocking buffer for 2 hours at room temperature. Cells were then counterstained with DAPI and mounted with ProLong© Diamond Anti-Fade Mountant (Thermo Fischer).

### Light microscopic autoradiography

Thick slices cultured under NGF tube diffusion were washed and incubated in serum free medium supplemented with 50 ng/mL ^125^I-NGF (specific activity 1320 Ci/mmol; final concentration 10 mCi/mL; Amersham, Buckinghamshire, England) for 2 hours at 37 °C. The specificity of the iodinated NGF binding was established by adding 50 μg/mL of unlabelled NGF. After being rinsed with cold culture medium, the slices were fixed in a 4% PFA solution for 48 hours, washed in PBS, osmicated, dehydrated in ethanol and embedded in Epon 812. Five µm sections were cut, coated with a monolayer of LM-1 emulsion (Amersham) and exposed for one month at 4 °C. They were developed with Kodak D-19 developer for 4 min, counterstained with 0.1% safranin, dehydrated and mounted in Eukitt.
